# “Silicon-On-Insulator”-Based Nanosensor for the Revelation of MicroRNA Markers of Autism

**DOI:** 10.3390/genes13020199

**Published:** 2022-01-22

**Authors:** Yuri D. Ivanov, Kristina A. Malsagova, Kristina V. Goldaeva, Tatyana O. Pleshakova, Ivan D. Shumov, Rafael A. Galiullin, Svetlana I. Kapustina, Ivan Y. Iourov, Svetlana G. Vorsanova, Stepan V. Ryabtsev, Vladimir P. Popov, Alexander I. Archakov

**Affiliations:** 1 Institute of Biomedical Chemistry, Pogodinskaya St. 10/8, 119121 Moscow, Russia; kristina.malsagova86@gmail.com (K.A.M.); goldaeva_1996@mail.ru (K.V.G.); t.pleshakova1@gmail.com (T.O.P.); shum230988@mail.ru (I.D.S.); rafael.anvarovich@gmail.com (R.A.G.); sveta.kapustina7.05@gmail.com (S.I.K.); alexander.archakov@ibmc.msk.ru (A.I.A.); 2 Mental Health Research Center, 117152 Moscow, Russia; ivan.iourov@gmail.com; 3 Veltischev Research and Clinical Institute for Pediatrics, Pirogov Russian National Research Medical University, Ministry of Health of Russian Federation, Taldomskaya St. 2, 125412 Moscow, Russia; svorsanova@mail.ru; 4 Center for Research of Social Systems, 354340 Sochi, Russia; Bob8bob@mail.ru; 5 Rzhanov Institute of Semiconductor Physics, Siberian Branch of Russian Academy of Sciences, 630090 Novosibirsk, Russia; popov@isp.nsc.ru

**Keywords:** nanosensor, autism, microRNA, nucleic acid detection

## Abstract

MicroRNAs (miRNAs), which represent short (20 to 22 nt) non-coding RNAs, were found to play a direct role in the development of autism in children. Herein, a highly sensitive “silicon-on-insulator”-based nanosensor (SOI-NS) has been developed for the revelation of autism-associated miRNAs. This SOI-NS comprises an array of nanowire sensor structures fabricated by complementary metal–oxide–semiconductor (CMOS)-compatible technology, gas-phase etching, and nanolithography. In our experiments described herein, we demonstrate the revelation of ASD-associated miRNAs in human plasma with the SOI-NS, whose sensor elements were sensitized with oligonucleotide probes. In order to determine the concentration sensitivity of the SOI-NS, experiments on the detection of synthetic DNA analogues of autism-associated miRNAs in purified buffer were performed. The lower limit of miRNA detection attained in our experiments amounted to 10^−17^ M.

## 1. Introduction

Autism spectrum disorders (ASD) represent developmental disorders characterized by short behaviour, dysfunction of sensory systems, and a tendency to stereotyped actions. In ASD, neuropsychiatric disorders usually manifest in the form of speech impairments, repetitive and/or compulsive behaviour, hyperactivity, anxiety, and difficulties with adaptation to new conditions and can also be accompanied by cognitive disorders [1].

As noted by the World Health Organization (WHO) [2], in the modern sense, ASD is an umbrella term covering such states as autism and Asperger syndrome. In addition, the USA Centers for Disease Control and Prevention (CDC) also attribute pervasive developmental disorder not otherwise specified (PDD-NOS) to ASD [3]. Worldwide, the prevalence of ASD was estimated to be 1:160—that is, 1 in 160 children is suffering from ASD [4]. At that, according to the WHO, this estimation is averaged, while the prevalence of ASD varies significantly [2,4]. The CDC performed a study on the prevalence of ASD among 8-year-old children residing within 11 ADDM sites in the USA, and this prevalence was reported to vary between 13.1 and 29.3 per 1000 children, while the overall prevalence of ASD was 16.8 per 1000 [5]. Since the data on the ASD prevalence reported by WHO and CDC differ considerably, one can conclude that the diagnosis of ASD represents an important problem of modern paediatrics. In general, the controversial points in the ASD diagnosis come down to several main aspects: the lack of a uniform diagnostic technique; the variation between the approaches to the understanding of the matter of ASD; and the vagueness of the diagnostic criteria. The high heterogeneity of the clinical presentation of ASD also makes its diagnosis difficult and ambiguous, particularly at the early stages [6]. On the one hand, all the above-mentioned facts lead to a significantly high number of individuals with undiagnosed ASD; on the other hand, they also cause an overdiagnosis of ASD in some regions.

To date, standardized tests (Childhood Autism Rating Scale, Modified Checklist for Autism in Toddlers, Revised, Autism Treatment Evaluation Checklist, Checklist for Autism Spectrum Disorder, Autism Diagnostic Observation Schedule, Autism Diagnostic Interview–Revised) are commonly used for the diagnosis of ASD. The principle of these tests consists in the following: a parent, a psychotherapist, a neurologist or a psychiatrist assesses the patient using an available scale. This assessment is performed on the basis of observations and questioning of the patient or his relatives. Obviously, using such an approach, the assessments of the one and the same patient by different doctors can vary considerably. Despite the developers of the tests claiming that the probability of the revelation of ASD with these tests is very high, it does not withdraw the need for reliable biomarkers of ASD and differential diagnosis of other disturbances of development. Accordingly, the revelation of reliable biomarkers of ASD will significantly facilitate the early diagnosis of ASD, providing priority support for the most vulnerable group.

The studies on the pathogenesis of ASD revealed that microRNAs (miRNAs) play a direct role in the occurrence of the disease [7,8]. MiRNAs participate in various cell processes—such as development, proliferation, differentiation, growth control, homeostasis, and apoptosis. MiRNAs form a class of short (20 to 22 nt) non-coding RNAs. MiRNAs serve as post-transcriptional regulators; they bind to the 3′ untranslated region of messenger RNAs (mRNAs), inhibiting the translation or degradation of mRNA [9].

In several recent studies, miRNAs were considered as promising molecular biomarkers of various pathologies in humans [10,11,12], and this makes the detection of miRNAs a relevant task. As regards ASD, a number of miRNAs (miRNA-125b, miRNA-132, miRNA-146a, miRNA-146b, miRNA-137, miRNA-134, miRNA-134-5p, miRNA-138-5p, miRNA-34a, miRNA-181c, miRNA-30d, miR-106a, miR-494, miR-19a, miR-19b, etc.) were reported to be connected with these disorders [13,14].

At present, several research groups have attempted to use mass spectrometry (MS) for the revelation of changes in metabolomic and proteomic profiles of ASD patients [15,16,17,18,19]. It should be emphasized that, to date, the lower limit of protein detection in plasma attainable by MS is relatively high, amounting to 1 pM to 1 nM [20,21]. This is obviously insufficient for the reliable detection of low- and ultra-low-abundant protein and nucleic acid markers of somatic and infectious diseases required for their early diagnosis. Thus, the development of novel highly sensitive methods for the early revelation of ASD biomarkers represents an important direction of modern biomedical research [22], and nanotechnology-based methods—such as the ones employing silicon nanowire-based nanosensors—are just the case. These nanosensors pertain to molecular detectors, since they allow one to detect target biomarker molecules at the single-charge level [23]. The principle of operation of the silicon nanowire-based nanosensor (NW NS), which is analogous to that of a field-effect transistor, is based on the registration of an electric current flowing through its nanowire (NW) sensor element. Namely, the adsorption of target biomarker molecules (which act as virtual gates in this respect) from the analysed sample onto the NW surface modulates the NW’s conductance, and the corresponding change in the electric current through the NW is registered in real time. NW NS allows one to detect a wide variety of targets—such as viral particles [24], proteins [25], and nucleic acids [26]—in real-time without introducing additional labels, attaining low (<10^−15^ M) limits of detection [27].

Herein, a “silicon-on-insulator” (SOI)-based NW NS (SOI-NS) has been employed for the detection of ASD-associated miRNAs isolated from plasma of ASD patients. The outer surface of the SOI NWs was sensitized with DNA oligonucleotide (oDNA) probes complementary to the target biomarker molecules, providing their biospecific capturing from the analysed samples. The successful application of such an oDNA-sensitized SOI-NW nanosensor for the detection of both the complimentary oDNA molecules in purified solutions and the target miRNAs isolated from a multicomponent biological matrix (plasma) at ultra-low concentrations down to 1.1 × 10^−17^ M has been experimentally demonstrated.

Further development of the proposed approach can allow us to develop an adequate diagnostic system for the early diagnosis of ASD on the basis of quantitative data obtained in the course of serological analysis. It is to be emphasized that this approach utilizes direct label-free, amplification-free detection of target biomarker molecules at ultra-low concentration in real time. These key features will allow one to perform correct and objective timely diagnosis of ASD in children.

## 2. Materials and Methods

### 2.1. Chemicals

Potassium phosphate monobasic (KH_2_PO_4_) and (3-aminopropyl)triethoxysilane (APTES) were purchased from Sigma-Aldrich (St. Louis, MO, USA). Hydrofluoric acid (HF) was purchased from Reakhim (Moscow, Russia). Isopropanol was purchased from Acros Organics (Geel, Belgium). 3,3′-dithiobis (sulfosuccinimidyl propionate) (DTSSP) was purchased from Pierce (Waltham, MA, USA). Ultrapure water (18.2 MΩ·cm) was obtained with a Simplicity UV deionizer (Millipore, Molsheim, France).

### 2.2. Oligonucleotides

The oDNA probes and the probe-complimentary oDNAs (used as model targets) were purchased from Evrogen (Moscow, Russia). Table 1 lists the nucleotide sequences of the oDNA probes employed in our experiments, and the sequences of both the model oDNA targets and the target miRNAs are listed in Table 2.

### 2.3. SOI-NS Fabrication

**Figure 1:**
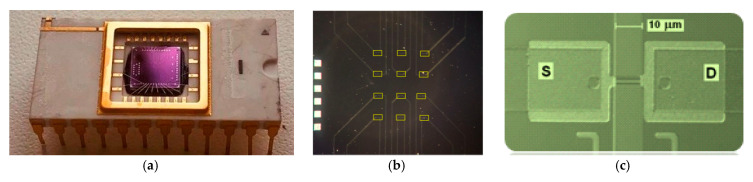
Photographic image of the entire “silicon-on-insulator”-based nanosensor (SOI-NS) sensor chip (**a**); optical image of the SOI-NS sensor chip with an array of 12 nanowire (NW) sensors (**b**); optical image of a single nanosensor (NS), which is located between source (S) and drain (D) contact areas (**c**). The central area of the chip with the array of NW NSs is to be in contact with the analysed liquid sample during measurements.

SOI-NS were fabricated using a technology similar to Smart Cut process, and comprised a hydrogen-induced transfer of Si layers onto a support layer. The differences of our technology from the Smart Cut process consisted of the following. The implantation hydrogen into the buried oxide (BOX) was not performed, and the top Si layer/BOX interface was bonded. Such an approach has allowed us to fabricate low-defect Si/SiO_2_ structures, providing stable parameters of the devices, in which these structures were utilized. The detailed description of the SOI structures formation technique was described elsewhere [29,30]. Namely, the SOI structures were fabricated as follows. NWs were fabricated by nanostructuring using SOI with n-type conductance. The initial total thickness of the SOI layers was 500 nm. In the process of fabrication, the thickness of the SOI layers was then reduced during multiple stages of thermal oxidation. As a result, the cut-off layer thickness and the BOX layer thickness amounted to 30 nm and 300 nm, respectively. BOX represents a gate oxide (SiO_2_) between the nanowire and the gate. After that, treatment with HF was performed in order to remove the natural oxide. The NW structures, located between the contact pads, were formed by optical litography structuring of the SOI. The next step was the formation of ohmic contacts by deposition of a poly-silicon layer with subsequent doping. Gas-plasma chemical etching and electron litography were employed for lateral structuring of the SOI layers. After metallization with subsequent contact pin assignment, crystal cut was performed, and this was the final step of the SOI structures fabrication. Since the fabrication of NWs represented a low-temperature process, it does not cause a degradation of SOI structures. The linear dimensions of each fabricated nanosensor were as follows: width *W* = 3000 nm, thickness *b* = 30 nm, and length *L* = 10 μm. The diameter of the sensitive area of the chip was ~2 mm. The isolation of individual NWs was performed by the formation of tetraethoxysilane-oxide during the pyrolysis of tetraethoxysilane by low-pressure chemical vapour deposition. Each sensor chip’s crystal bore an array of 12 individual NW sensors, arranged in pairs, and was integrated into a standard microcircuit frame as displayed in Figure 1.

### 2.4. Sensitization of the Sensor Chip Surface

In order to clean the sensor chip and water. The clean surface of the chip was rinsed with an HF:CH_3_OH (1:50) solution for 30 s in order to remove the native oxide layer formed on the surface during storage of the sensor chip. After that, the sensor chip was treated in a UV Ozone Cleaner–ProCleaner™ Plus device (Ossila Ltd., Sheffield, UK) in order to form hydroxyl groups on the sensor surface [31]. Then, the surface of the sensor chip was subsequently modified by forming an APTES layer on it by vapour deposition method described elsewhere [32]. Namely, the APTES layer was formed by the 20 h incubation of the sensor chip in APTES vapour at room temperature. The so-formed APTES layer should be of a definite (0.9 nm to 1.2 nm) thickness [32].

**Figure 2:**
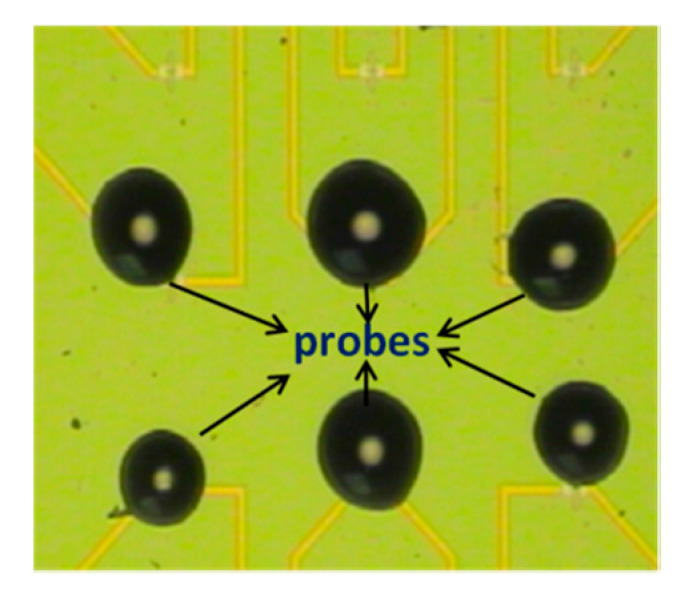
Optical image of 3-nL droplets dispensed onto individual nanosensors.

oDNA molecular probes were covalently immobilized onto the APTES-modified surface of the sensor chip via DTSSP crosslinker using the technique reported in our previous papers [33,34,35,36,37]. The concentration of the oDNA probes in the immobilization solution, prepared using 50 mM potassium phosphate buffer (pH 7.4), was 1 μM. The oDNA probes were immobilized on the surface of individual nanosensors by precisely dispensing ~2 nL microdrops of the immobilization solutions onto the individual nanosensors with an iONE spotter (M2-Automation GmbH, Berlin, Germany) equipped with a piezo-driven PDMD micro-dispenser (M2-Automation GmbH, Berlin, Germany). The microdrops of the oDNA solutions were incubated on the NS surface for 30 min at 5 °C in a humid chamber and then washed away with deionized water and dried under nitrogen. Figure 2 displays optical image of the microdrops on the sensor chip surface.

### 2.5. Electrical Biosensor Measurements

**Figure 3:**
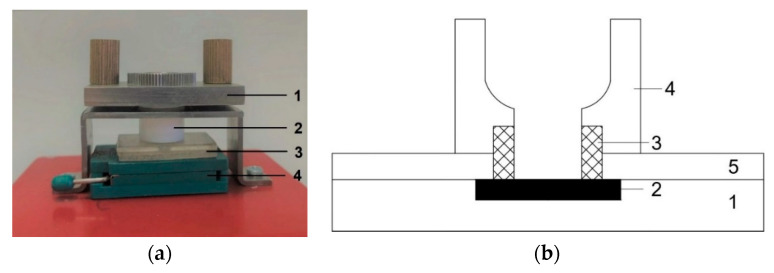
Analytical unit of the “silicon-on-insulator”-based nanosensor. (**a**) Photographic image of the analytical unit. Numbers indicate the main elements of the unit: 1—pressing mechanism; 2—measuring cell; 3—sensor chip with a polystyrene cover; 4—sensor chip holder. (**b**) Schematic of the analytical unit without the pressing mechanism. Numbers indicate the elements of the unit: 1—sensor chip; 2—sensor chip crystal bearing an array of nanowires; 3—polyvinyl chloride gasket; 4—measuring cell; 5—polystyrene cover.

Figure 3 displays a photographic image (Figure 3a) and a schematic (Figure 3b) of an analytical unit of the SOI-NS, which was employed for the electrical biosensor measurements.

The sensor chip was fixed in a chip holder and a 1-mL polytetrafluoroethylene measuring cell was placed onto the sensor chip so that the latter served as the cell bottom. A sterile polyvinyl chloride gasket was placed between the measuring cell and the sensor chip surface. A polystyrene cover was placed onto the chip in order to center the gasket, preventing its displacement to the sensitive area of the chip. The measuring cell was additionally fixed with a pressing mechanism, which provided an even, leak-tight adherence of the gasket to the chip surface. This construction represented the analytical unit of the biosensor. The cell was equipped with a stirrer, and the stirring rate was 3000 rpm.

The measurements were performed with a 10-channel data collection and storage system (Agama+ JSC, Moscow, Russia). The dependencies of the drain-source current on gate voltage *I_ds_*(*V_g_*) (current-voltage characteristics, CVCs) were recorded in buffer solution. The gate voltage *V_g_* was varied between 0 and 60 V, while the drain-source voltage *V_ds_* = 0.1 V was constant. The SOI structures’ substrate served as a gate.

In the experiments on the detection of the model target oDNAs (CS), a 50 μL volume of oDNA-containing solution was added to 150 μL of measurement buffer in the measuring cell. In the experiments on the revelation of target miRNAs isolated from plasma samples, a 7 μL volume of miRNA-containing sample was added to the 100 μL of the measurement buffer in the cell. Throughout the biosensor measurements, the time dependencies of the current *I_ds_*(*t*) were recorded for each individual NW in subthreshold mode at *V_g_* = 50 V and *V_d_*_s_ = 0.1 V.

Once again, the above-described technique of the sensitisation of individual nanosensors was employed. Thus, the NS array contained both working NSs and reference NSs within one and the same sensor chip. The surface of the working NSs was sensitized with immobilized oDNA probes, while the surface of the reference NS was devoid of covalently immobilized biomolecules.

To account for a non-specific adsorption onto the NS surface, a differential signal Δ*I_ds_(t)* was calculated in the following way. The registered changes in the level of the current signal *I_ds_* from each NW were normalized to 1 by dividing their value by the initial value of the current. Then, the values obtained in the blank experiment performed with a pure nucleic acid-free buffer as the sample solution instead of model oDNA solution (or 75% v/v aqueous ethanol instead of miRNA-containing sample) were subtracted from those obtained upon the analysis of the solution containing target molecules. After that, the difference between the normalized signal from the working NS and that from the reference NS was calculated. The resulting Δ*I_ds_(t)* dependencies were presented in the form of sensogram curves.

### 2.6. Preparation of Test Solutions Containing Known Concentrations of Model Target oDNAs

Test solutions containing known concentrations of model target oDNAs in the range from 1 aM to 10 fM were prepared by the sequential 10-fold dilution of 100 µM solution of the corresponding model oDNAs in 50 mM potassium phosphate buffer (pH 7.4) with working 1 mM potassium phosphate buffer (pH 7.4). On each dilution step, the solution was incubated in a Thermomixer Comfort shaker (Eppendorf, Germany) for 0.5 h at 10 °C and 600 rpm. The so-prepared test solutions were immediately used in biosensor experiments.

### 2.7. Plasma Samples

Plasma samples were obtained from patients with diagnosed ASD. The samples were provided by the Mental Health Research Center and Laboratory of Molecular Cytogenetics of Neuropsychiatric Diseases, Veltischev Clinical Pediatric Research Institute, Pirogov Russian National Research Medical University (Moscow, Russia). Written informed consents were obtained from all study participants. Table 3 lists the information about the plasma samples studied herein.

The plasma samples were obtained in the following way. Firstly, blood samples were taken from the cubital vein before treatment on an empty stomach. The blood samples were collected into specialized containers with 3.8% CH_3_COONa (IMPROVACUTER, Guangzhou Improve Medical Instruments Co., Ltd., Guangzhou, China) and centrifuged (3000 rpm, 6 min, room temperature). The so-obtained 500-μL plasma samples were then placed into dry Eppendorf-type test tubes, frozen to −80 °C, and stored until their use in the experiments.

MiRNAs were isolated from the studied plasma samples with an ExtractRNA (Evrogen, Moscow, Russia) according to the protocol provided by the manufacturer. The so-isolated miRNA samples were diluted with 75% ethanol [38]. In the blank experiments, pure buffer solution was treated in the same way.

## 3. Results

### 3.1. Detection of Model Target oDNAs in Purified Solutions

**Figure 4:**
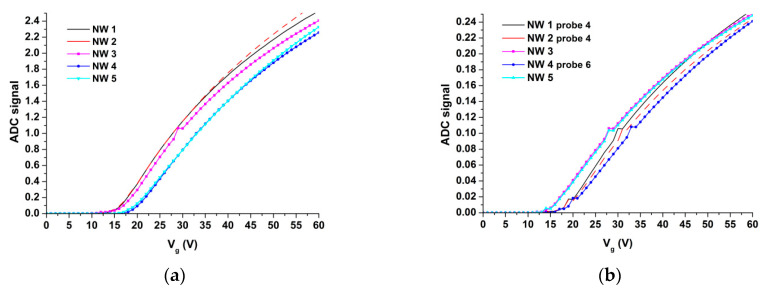
Typical current-voltage characteristics recorded for two groups of five individual nanosensors (NSs); each group was located on a different sensor chip: SOI-NS#1 (**a**) and SOI-NS#2 (**b**). Experimental conditions: NSs No. 3, No. 4, and No. 5 with n-type conductance were sensitized with covalently immobilized oDNA probes #1, #2, and #4, respectively (**a**); NSs No. 1, No. 2, and No. 4 with n-type conductance were sensitized with covalently immobilized oDNA probes #4, #4, and #6, respectively (**b**); 1 mM potassium phosphate buffer (pH 7.4); *V_ds_* = 0.1 V.

Firstly, the current-voltage characteristics (CVCs) of the nanosensors were recorded in order to determine optimal *V_g_* value. Figure 4 displays typical CVC curves, recorded for the nanosensors located on two different sensor chips.

Based on the CVCs obtained, the optimal *V_g_* value was determined to be 50 V. At that, from the comparison of Figure 4a,b, one can observe that the overall level of the current signal obtained for SOI-NS#1 sensor chip is approximately 10 times higher than that for SOI-NSs#2 due to the fact that the fabricated sensor chips initially have different technical characteristics—including the conductance level. At present, we are performing work in order to standardize the technology. The 50 V *V_g_* value was selected according to the following considerations. In fact, the NW on the SOI substrate represents a dual-gate transistor. In this transistor, one (local) gate is a particle adsorbed onto the NW surface; the other gate (back gate) is the substrate of the SOI structure. This is why the signal from the biosensor can alter depending on the concentration of charged particles adsorbed onto the NW surface. At a lower gate voltage, the signal from the biosensor can drop down to minimum values (and even down to the noise level), thus hindering its registration.

In the experiments on the SOI-NS-based detection of model target oDNAs in purified test solutions, the time dependencies of the current Δ*I_ds_(t)* were recorded in real time at fixed *V_g_* = 50 V. Moreover, 1 mM potassium phosphate buffer was employed as the working medium in order to avoid the Debye screening problem, since at such salt concentration, the Debye length λ_D_ amounts to ~6.65 nm, being sufficient for the successful registration of (probe oDNA)/(target oDNA) complex formation on the NS surface [39,40].

**Figure 5:**
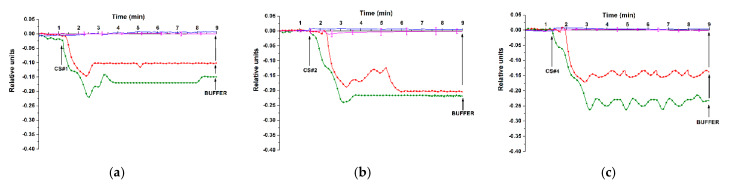
Typical sensogram curves recorded throughout the real-time detection of the CS#1, CS#2, and CS#4 model target oDNAs, which represent synthetic analogues of miR-106a-5p (**a**), miR-106b-5p (**b**), and miR-494-5p (**c**), respectively, at concentrations from 1.1 × 10^−18^ M to 1.1 × 10^−16^ M. Experimental conditions: SOI-NS#1 sensor chip with nanosensors of n-type conductance No. 3 (**a**), No. 4 (**b**), No. 5 (**c**), which were sensitized with covalently immobilized oDNA probe #1 (**a**), probe #2 (**b**), and probe #4 (**c**), respectively; 1 mM potassium phosphate working buffer (pH 7.4); *V_g_* = 50 V; *V_ds_* = 0.1 V. Target oDNAs concentration was 1.1 × 10^−18^ M (blue), 1.1 × 10^−17^ M (red), 1.1 × 10^−16^ M (green), and blank experiment with oDNA-free working buffer (magenta, the number of technical replicates *n* = 3). For the blank experiment, the data are presented with standard deviation error ± σ. Arrows indicate the addition of the test solution and the wash with pure working buffer.

For the detection of model target oDNAs in test solutions at ultra-low concentrations (namely, within the 1.1 × 10^−18^ M to 1.1 × 10^−16^ M concentration range), the SOI-NS#1 oDNA-sensitized sensor chip was employed. The solutions of either CS#1, CS#2, or CS#4 target oDNAs were added to the working 1 mM potassium phosphate buffer in the measuring cell. In order to account for the non-specific binding, an NS devoid of immobilized oDNA probes and located within the same NS array was used as a reference NS. Figure 5 displays typical sensogram curves recorded throughout the real-time detection of the CS#1, CS#2, and CS#4 model target oDNAs, which represent synthetic analogues of miR-106a-5p, miR-106b-5p, and miR-494-5p, respectively, at concentrations from 1.1 × 10^−18^ M to 1.1 × 10^−16^ M.

In Figure 5, the blue sensogram shows the signal obtained upon the analysis of 1.1 × 10^−18^ M oDNA solution. This sensogram clearly indicates the absence of any change in the signal level at such a low target oDNA concentration. At higher oDNA concentrations (1.1 × 10^−17^ M, red sensogram; 1.1 × 10^−16^ M, green sensogram), a decrease in the current flowing through the respective NSs is observed. The sensograms shown in Figure 5 clearly indicate that, within the concentration range studied, the higher the concentration of the target oDNA is, the greater is the amplitude of the decrease in the current signal.

**Figure 6:**
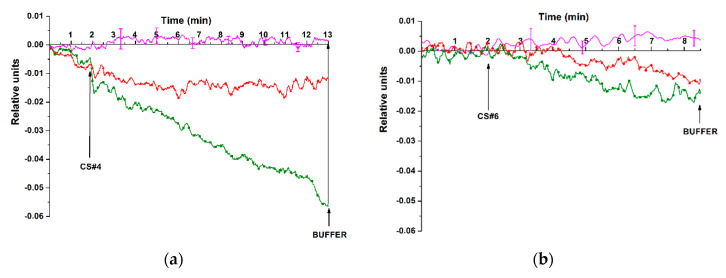
Typical sensogram curves recorded throughout the real-time detection of the CS#4 and CS#6 model target oDNAs, which represent synthetic analogues of miR-494-5p (**a**), and miR-15b-5p (**b**), respectively, at concentrations of 1.1 × 10^−15^ M and 1.1 × 10^−14^ M. Experimental conditions: SOI-NS#2 sensor chip with nanosensors of n-type conductance No. 1 (**a**) and No. 5 (**b**), which were sensitized with covalently immobilized oDNA probe #4 (**a**) and probe #6 (**b**), respectively; 1 mM potassium phosphate working buffer (pH 7.4); *V_g_* = 50 V; *V_ds_* = 0.1 V. Target oDNAs concentration was 1.1 × 10^−15^ M (red), 1.1 × 10^−14^ M (green), and blank experiment with oDNA-free working buffer (magenta, the number of technical replicates *n* = 3). For the blank experiment, the data are presented with standard deviation error ± σ. Arrows indicate the addition of the test solution and the wash with pure working buffer.

For the detection of model target oDNAs in the test solutions at higher concentrations within the range from 1.1 × 10^−15^ M to 1.1 × 10^−14^ M, another sensor chip, SOI-NS#2, was used. The test solutions containing known concentrations of either CS#4 or CS#6 model target oDNAs (which represent synthetic analogues of miR-494-5p and miR-15b-5p, respectively) were added to the working buffer in the measuring cell. This sensor chip also contained oDNA-probes-free reference NS in order to account for the non-specific adsorption. Figure 6 displays typical sensograms recorded during the detection of CS#4 and CS#6 target oDNAs in purified working buffer at concentrations ranging from 1.1 × 10^−15^ M to 1.1 × 10^−14^ M.

The sensograms shown in Figure 6 indicate a decrease in the electric current flowing through the probe oDNA-sensitized NSs upon the detection of model target oDNAs at concentrations from 1.1 × 10^−15^ M to 1.1 × 10^−14^ M. At that, for CS#4 oDNA at 1.1 × 10^−15^ M concentration, it was shown that upon its repeated detection, the maximum change in the signal level amounted to ~20% (Figure 6a, red sensogram). The decrease in the signal upon the detection of the model target oDNAs at both ultra-low concentrations (using SOI-NS#1 sensor chip) and low concentrations (using SOI-NS#2 chip) can be caused by the influence of a negative charge of the target oDNAs. This, in turn, induces an increase in the electric charge density in the vicinity of the sensor surface during the biospecific capturing of the oDNA molecules from their solution onto the sensor surface. Such an increase in the negative charge is expected to cause a decrease in the electric current through the NS. The sensograms shown in Figure 5; Figure 6 clearly indicate a decrease in the amplitude of the signal with the decreasing concentration of the model target oDNAs from 10^−16^ M to 10^−18^ M and from 10^−14^ M to 10^−15^ M, respectively. It should be emphasized that in the blank experiments, either no change in the signal was registered or the amplitude of this change was negligibly small (magenta-coloured sensograms in Figure 5; Figure 6). The results obtained confirm the occurrence of a biospecific interaction between the sensor-immobilized oDNA probes and their complimentary model target oDNAs. In addition, it should be emphasized that the minimum detectable oDNA concentration amounted to 1.1 × 10^−17^ M for three different model target oDNAs, which represent synthetic analogues of ASD-associated miR-106a-5p, miR-106b-5p, and miR-494-5p, respectively.

### 3.2. Biospecific Detection of miRNAs Isolated from Blood Plasma

**Figure 7:**
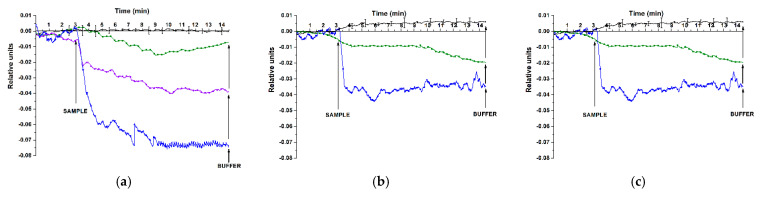
Typical sensograms recorded in the experiments on the analysis of miRNAs isolated from plasma samples. Experimental conditions: SOI-NS#1 sensor chip with nanosensors sensitized with probe #1 (**a**), probe #2 (**b**), and probe #4 (**c**) oligonucleotide probes. The RNA samples were isolated from the plasma of a healthy individual (sample No.#1; black) or from the plasma of ASD patients (sample No. #2, blue; sample No. #3, green; sample No. #7, violet); 1 mM potassium phosphate working buffer; *V_g_* = 50 V, *V_ds_*= 0.1 V, total volume of the solution in the cell 107 µL. For the control experiment (black), the data are presented with standard deviation error ± σ, the number of technical replicates *n* = 3. Arrows indicate the addition of miRNA samples and wash with pure working buffer.

**Figure 8:**
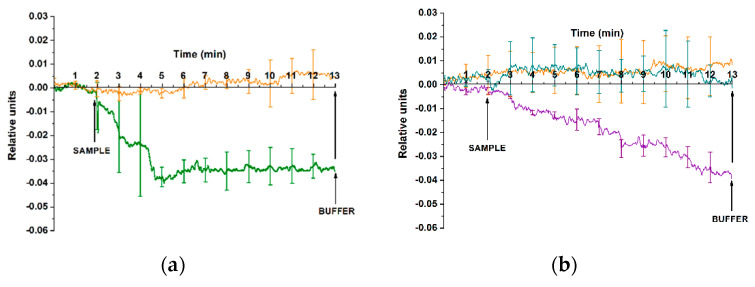
Typical sensograms recorded in the experiments on the analysis of miRNAs isolated from plasma samples. Experimental conditions: SOI-NS#2 sensor chip nanowires sensitized with probe #4 (**a**), and probe #6 (**b**) oligonucleotide probes; the number of technical replicates *n* = 3. The miRNA samples were isolated from the plasma of healthy individuals (sample No.#4, orange, **a**; sample No. #8, turquoise, **b**) or from plasma of ASD patients (sample No. #3, green, **a**; sample No. #11, purple, **b**); 1 mM potassium phosphate working buffer; *V_g_* = 50 V, *V_ds_* = 0.1 V, total volume of the solution in the cell 107 µL. Arrows indicate the addition of miRNA samples and wash with pure working buffer.

Figure 7; Figure 8 display typical sensograms recorded in the experiments on the analysis of miRNAs isolated from plasma samples obtained from either ASD patients or from healthy individuals using SOI-NS#1 and SOI-NS#2 sensor chips, respectively.

The sensograms shown in Figure 7 indicate a decrease in the conductance of the oDNA-sensitized NSs of SOI-NS#1 sensor chip after the addition of miRNA samples isolated from plasma of ASD patients—similar to the case with the model target oDNAs (Figure 5). Namely, upon the analysis of miRNA isolated from plasma samples Nos. #2 and #3, a decrease in the current flowing through NSs sensitized with probe#1, probe#2, and probe#4 oDNAs was observed (Figure 7a–c). Moreover, in the case of NS sensitized with probe#1 oDNA (whose surface is complementary to the synthetic analogue of miR-106a-5p), the current signal changed upon the addition of miRNA isolated from plasma sample #7. This is opposed to the situation observed in the control experiments with miRNA isolated from the sample of a healthy individual (sample #1), when the signal either did not change or changed insignificantly (Figure 7a–c, black).

The sensograms shown in Figure 8 indicate a decrease in the conductance of oDNA-sensitized NSs of SOI-NW#2 sensor chip after the addition of miRNA samples isolated from plasma of patients with confirmed ASD (samples Nos. #3, #11). This was similar to the case with the model target oDNAs (Figure 6). Namely, upon the analysis of miRNA samples isolated from plasma of patients with confirmed ASD diagnosis (sample No. #3), a decrease in the current flowing through the NS sensitized with probe #4 oDNA (complementary to the synthetic analogue of miR-494-5p); at that, a decrease in the signal from the NS sensitized with probe #6 (complementary to the synthetic analogue of miR-15b-5p) was observed upon the analysis of miRNA samples isolated from plasma of patients with confirmed ASD diagnosis (sample No.#11). On the contrary, in the control experiments with the miRNA isolated from the sample of healthy individuals, the change in the signal was insignificant (sample No.#4, orange; sample No.#8, turquoise).

**Figure 9:**
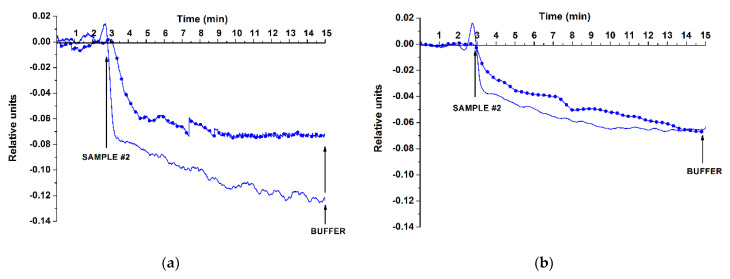
Typical sensograms recorded upon the analysis of one and the same miRNA sample isolated from plasma of a patient with confirmed ASD diagnosis (sample No #2). Experimental conditions: SOI-NW#1 sensor chip; NSs of n-type conductance sensitized with probe #1 (**a**) and probe #4 (**b**); 1 mM potassium phosphate buffer; *V_g_* = 50 V; *V_ds_* = 0.1 V, total volume of the solution in the cell 107 µL. The experiment was performed on different days (1st day of the experiment, circles ●; 2nd day of the experiment, solid lime ▬). Arrows indicate the addition of miRNA samples, and wash with pure working buffer.

Figure 9 displays the typical sensograms recorded upon the analysis of one and the same miRNA sample isolated from the plasma of a patient with confirmed ASD diagnosis (sample No. #2) in order to illustrate the reproducibility of the results obtained with our SOI-NS and the efficiency of the regeneration of the sensor surface.

The experiment on the analysis of one and the same miRNA sample isolated from plasma of a patient with confirmed ASD diagnosis was being performed for two working days. At the end of the first working day (after the experiments), and at the beginning of the next working day, the surface of SOI-NW#2 sensor chip was washed with ultrapure water (72 °C, 50 mL) [41]. The analysis of the data obtained in these experiments has indicated that upon repeated analysis of one and the same sample, changes in the signal from the NS sensitized with probe #1 (Figure 9a) make up ~40%, while the signal was virtually unchanged in the case with the NS sensitized with probe #4 (Figure 9b). Thus, the previously developed technique for the regeneration of the SOI-NS sensor surface allows for the repeated use of the SOI-NS sensor chip in the analysis of biological fluids [33,34,35,36,37].

## 4. Discussion

The timely diagnosis of ASD is important for personalized treatment. In this respect, the diagnosis of ASD at the early stages is particularly important, since it can prevent further development of the disease to more severe forms. In this connection, it should be borne in mind that children with ASD can learn to mask the symptoms as they develop intelligence and awareness, thus impairing the diagnosis accuracy [42]. In addition, an accurate and clear ASD diagnosis can provide necessary clinical support to the patients’ parents.

The present study was aimed at the development of a label-free approach to the revelation of ASD-associated miRNAs (miR-106a-5p, miR-106b-5p, miR-494-5p, miR-15b-5p) [13,28] in the blood of ASD patients. In the first step, the experiments on the detection of model target oDNAs in purified test solutions with known oDNA concentrations have been performed in order to determine the lowest concentration of target oligonucleotides detectable with the SOI-NS. On this step, oDNA-free buffer was used in blank experiments instead of oDNA solutions. The model target oDNAs (designated as CS#1; CS#2; CS#4; CS#6) represented synthetic oligonucleotide analogues of the target miRNAs [13,28]. In the second step, samples of miRNA isolated from plasma of patients with confirmed ASD diagnosis were analysed, while miRNA samples isolated from the plasma of healthy individuals were used as control samples.

In order to provide biospecific capturing of the target oDNAs and miRNAs, the surface of individual nanosensors has been sensitized with oDNA molecular probes (designated as “probe 1”, “probe 2”, “probe 4”, and “probe 6”), whose sequences were complementary to those of the target biomolecules.

The effect of an electric field—namely, the penetration of the field into a liquid medium—is quantitatively characterized with the Debye screening length (*λ_D_*). Physically, the Debye length corresponds to the characteristic distance, over which the electric field penetrates into the liquid. In order to avoid problems associated with the influence of the Debye screening, the electrical detection experiments were performed with the use of a low-salt (1 mM) buffer. At such a low concentration of buffer salts, *λ_D_* amounts to ~6.65 nm, and this is sufficient for the registration of a nucleic acid on the NW surface [40]. At higher concentrations of buffer salts, the electric double layer becomes shorter than the length of the DNA duplex (*λ_D_* = 6.65 nm for 1 mM phosphate buffer), thus leading to a decrease in the detection sensitivity, since the hybridization partially occurs outside the electric double layer region [40].

The experiments on the detection of synthetic oDNA analogues of miR-106a-5p, miR-106b-5p, and miR-494-5p with SOI-NW#1 have shown that three of these synthetic oDNAs—CS#1, CS#2, and CS#4—are detectable with our nanosensor. The lowest concentration of the model target oDNA, detectable with SOI-NW#2 in purified buffer, amounted to 1.1 × 10^−17^ M. It should be noted that CS#1, CS#2, and CS#4 oDNAs did not form complexes with non-complementary NS-immobilized probes. This indicates the biospecificity of the target model oDNA detection.

Experiments on the detection of the model target oDNAs, which represented synthetic analogues of miR-494-5p and miR-15b-5p with the use of an SOI-NW#2 sensor chip, have shown that two of the model oDNAs used, CS#4 and CS#6, can be well detected at concentrations from 10^−15^ M to 10^−14^ M.

In the second step of our research, the experiments with samples of miRNAs isolated from plasma were performed in order to determine whether our SOI-NS is applicable for the analysis of real biological samples. The applicability of the approach developed herein for the analysis of real biological samples has been demonstrated.

Upon the detection of miRNAs isolated from plasma of patients with confirmed ASD diagnosis using SOI-NW#1 sensor chip, the absolute value of the registered signal was approximately 5 times lower than that in the experiment with 1.1 × 10^−15^ M model target oDNA solution. In parallel, in the case of the SOI-NW#2 sensor chip, this value was either 2 times greater (probe 6) than or equal to (probe 4) that obtained for the 1.1 × 10^−14^ M model target oDNA solution. Regarding the concentration of the target miRNAs in the analysed plasma samples, it should be taken into account that its estimation can only be performed roughly due to the losses of target miRNAs’ molecules on the preanalytical (sample preparation) step. According to the data obtained throughout our experiments, the concentration of miR-106a-5p, miR-106b-5p, miR-494-5p, and miR-15b-5p in plasma samples of ASD patients is estimated to be of the order of 10^−17^ to 10^−16^ M.

In our opinion, the detection limit attained herein with our SOI-NS is considerably low. Despite real-time polymerase chain reaction (rtPCR) allowing one to detect lower concentrations of target nucleic acids, it is extremely sensitive to sample contamination due to the use of amplification reaction, which can lead to false results—as opposed to the amplification-free method proposed herein. This circumstance is quite important and must be taken into account in the development of diagnostic systems.

Herein, it has been demonstrated that SOI-NS allows one to reveal an increased level of ASD-associated miRNAs (miR-106a-5p, miR-106b-5p, miR-494-5p) in samples of children suffering from ASD in comparison with that in control samples. At that, our biosensor-based approach is label-free, and the measurements are performed in real time.

As noted by Tonacci et al. [13], the development of ASD is accompanied by the miR-15b-5p downregulation. In our experiments, however, this miRNA has been revealed in plasma samples studied. In order to clarify this result, additional research using both the nanosensor-based detection and determination of miRNA expression by polymerase chain reaction (PCR) and sequencing is possibly required.

A highly reliable blood test could meet the requirements for ASD diagnosis. In perspective, the approach developed herein can be used in clinical practice for the diagnosis of ASD, particularly upon the appearance of the first symptoms of the disease appear, as well as throughout the following treatment. Further research is, however, required in order to perform validation, the refinement of results, and the reduction of false positive results.

## 5. Conclusions

SOI-NS sensor chips, bearing an array of 12 sensor elements (nanowires), were fabricated by a complementary metal–oxide–semiconductor (CMOS)-compatible technology, using gas-phase etching and litography. The use of the so-fabricated SOI-NS for the label-free detection of target nucleic acids at low (down to 10^−17^ M) concentrations in real time has been experimentally demonstrated with the example of synthetic oDNA analogues of ASD-associated miRNAs. Moreover, the applicability of such an SOI-NS for the diagnosis of ASD by the analysis of the content of marker miRNAs, isolated from the plasma of ASD patients, has also been demonstrated. It has been found that our SOI-NS allows one to detect a change in the miRNA level in ASD patients—in comparison with healthy individuals. The results obtained herein are in agreement with the literature data on the increase in the level of marker miRNAs in ASD. Thus, in perspective, the approach developed herein can serve as a fundamental platform for the development of novel methods of screening diagnostics of patients with ASD.

## Figures and Tables

**Table 1:** The nucleotide sequences of the oDNA probes.

No. Probe	Sequence oDNA Probes
probe#1	(NH_2_)-T_10_CTACCTGCACTGTAAGCACTTTT
probe#2	(NH_2_)-T_10_ATCTGCACTGTCAGCACTTTA
probe#4	(NH_2_)-T_10_AGAGAAGACAACACG GACAACCT
probe#6	(NH_2_)-T_10_TGTAAACCATGATGTGCTGCTA

**Table 2:** The nucleotide sequences of the model oDNA targets and target miRNAs.

oDNA	Model Target oDNA Sequence	Target miRNA Sequence [Ref.]
*CS#1	AAAAGTGCTTACAGTGCAGGTAG	miR-106a-5p [13,28]
CS#2	TAAAGTGCTGACAGTGCAGAT	miR-106b-5p [13,28]
CS#4	AGGTTGTCCGTGTTGTCTTCTCT	miR-494-5p [13,28]
CS#6	TAGCAGCACATCATGGTTTACA	miR-15b-5p [13,28]

*CS, Complementary Sequence.

**Table 3:** Clinical and morphological characteristics of plasma samples obtained from ASD patients and from healthy individuals.

Sample No.	Age	Sex	Diagnosis
#1	49	female	healthy individual
#2	9	male	ASD
#3	20	male	ASD
#7	7	male	ASD
#4	33	female	healthy individual
#8	5	male	healthy individual
#11	6	male	ASD

## Data Availability

Correspondence and requests for materials should be addressed to Y.D.I.
